# MiR-421 Binds to PINK1 and Enhances Neural Stem Cell Self-Renewal *via* HDAC3-Dependent FOXO3 Activation

**DOI:** 10.3389/fcell.2021.621187

**Published:** 2021-07-20

**Authors:** Jiaoying Jia, Ming Wang, Min Liu, Zhigang Tan, Yan Cui, Mengqiang Yu

**Affiliations:** Department of Neurosurgery, The Second Xiangya Hospital of Central South University, Changsha, China

**Keywords:** PINK1, HDAC3, FOXO3, neural stem cell, self-renewal, ischemic brain injury, microRNA-421

## Abstract

Dysfunctions of neural stem cells (NSCs) often lead to a variety of neurological diseases. Thus, therapies based on NSCs have gained increasing attention recently. It has been documented that microRNA (miR)-421 represses the autophagy and apoptosis of mouse hippocampal neurons and confers a role in the repair of ischemic brain injury (IBI). Herein, we aimed to illustrate the effects of miR-421 on NSC self-renewal. The downstream factors of miR-421 were predicted initially, followed by gain- and loss-of-function assays to examine their effects on NSC self-renewal. Immunoprecipitation and dual luciferase assays were conducted to validate the interaction among miR-421, PTEN-induced putative kinase 1 (PINK1), HDAC3, and forkhead box O3 (FOXO3). A mouse model with IBI was developed to substantiate the impact of the miR-421/PINK1/HDAC3/FOXO3 axis on NSC self-renewal. The expression of miR-421 was downregulated during differentiation of human embryonic NSCs, and miR-421 overexpression accelerated NSC self-renewal. Besides, miR-421 targeted PINK1 and restricted its expression in NSCs and further suppressed HDAC3 phosphorylation and enhanced FOXO3 acetylation. In conclusion, our data elucidated that miR-421 overexpression may facilitate NSC self-renewal through the PINK1/HDAC3/FOXO3 axis, which may provide potential therapeutic targets for the development of novel therapies for IBI.

## Introduction

Despite intensive research for many years, management for disorders of the nervous system, including ischemic brain injury (IBI), trauma-induced nerve injuries, and neurodegenerative diseases, remains poorly developed (Ma et al., 2018). Since it is hard for nerve cells to self-heal or self-renew spontaneously, therapeutics based on the neural stem cells (NSCs) have obtained increasing attention in recent years (Lang and Obeso, 2012; Giusto et al., 2014). Although NSCs have a role to confer in brain development and neurogenesis maintenance in specific brain areas (Bertolini et al., 2019), many tricky problems need to be solved before applying the NSC-based therapy for neurological diseases. A major challenge for NSC application is that the inflammation following brain injury is not optimal for the proliferation or differentiation of transplanted NSCs (Yang et al., 2014). Therefore, it is urgent to investigate the molecular mechanism underlying NSC renewal in order to facilitate the development of NSC-based therapeutic regimens for the treatment of nervous system diseases.

Interestingly, evidence exists supporting that microRNA (miR)-421 is highly expressed in the amygdala and shows the potential for affecting the repair after brain injury (Yang et al., 2014). Moreover, overexpression of miR-421 restricts the autophagy and apoptosis of hippocampal neurons (Wen et al., 2018). Thus, we speculate that miR-421 may also play a role in the self-renewal of NSCs. Additionally, miR-421 can target and inhibit PTEN-induced putative kinase 1 (PINK1) (Wang et al., 2015), and PINK1 was identified as a differentially expressed gene in our prediction analysis. PINK1, a mitochondria-targeted serine/threonine kinase, enhances risk of Parkinson’s disease through a dominant-negative mechanism (Gandhi and Plun-Favreau, 2017). Meanwhile, PINK1 deficiency may lead to defects in GFAP-positive astrogliogenesis involved in brain development and NSC differentiation (Choi et al., 2016a). Hence, miR-421 may affect the renewal and differentiation of NSCs by regulating PINK1 expression.

Furthermore, PINK1 can restrict dopaminergic neuronal cell death by increasing histone deacetylase3 (HDAC3) phosphorylation (Choi et al., 2015). HDACs can affect the expression of downstream genes by removing acetyl groups, which is critical for chromatin remodeling in brain development and neural cell differentiation and death (Tiwari et al., 2014). It has also been reported that HDAC3 is upregulated after axonal injury (Pelzel et al., 2010). Moreover, HDAC3 can form a complex with forkhead box O3 (FOXO3) to reduce the degree of FOXO3 acetylation (Zhang et al., 2017). The FOXO family, the famous transcription factors, is a pivotal mediator of stem cells and implicated in aging and cellular homeostasis (Martins et al., 2016). Previous studies have illuminated that the FOXOs, particularly FOXO3, may contribute to NSC homeostasis in mice (Paik et al., 2009; Renault et al., 2009). The research of Audesse et al. (2019) has highlighted that FOXO3 directly participates in the regulation of autophagy genes network in NSCs. In this study, we hypothesize that miR-421 could affect the process of NSC self-renewal by mediating PINK1, acting in association with regulation of HDAC3/FOXO3.

## Materials and Methods

### Ethics Statement

Animal experiments were approved by the Animal Care and Use Committee of The Second Xiangya Hospital of Central South University and performed in accordance with *Guide for the Care and Use of Laboratory Animals* published by the US National Institutes of Health.

### Microarray-Based Gene Expression Profiling

NSC-related gene expression datasets GSE143388 and GSE134688 were retrieved from the Gene Expression Omnibus (GEO) database^1^. Gene expression data were extracted from 12 NSC samples in the GSE143388 dataset and 70 NSC samples in the GSE134688 dataset for correlation analysis.

### Cell Isolation, Culture, and Transfection

Primary NSCs were obtained from human embryos (E14.5) (Shanghai Jiesijie Laboratory Animal Co., Ltd., Shanghai, China). NSCs were cultured in the NSC basal medium containing a NeuroCult proliferation supplement (Stemcell Technologies, Vancouver, BC, Canada), 1% N2 (Gibco, Rockville, MD, United States), 20 ng/ml basic fibroblast growth factor (BGF; R&D Systems, Minneapolis, MN, United States), 20 ng/ml epidermal growth factor (EGF), 1% penicillin, and 1% streptomycin (Sigma-Aldrich Chemical Company, St. Louis, MO, United States). Cells were maintained in plastic flasks without coated tissues at 37°C in a humidified atmosphere with 5% CO_2_, and the culture medium was renewed every 3 days. Subsequently, the abovementioned NSCs were dissociated using StemPro Accutase cell dissociation reagent (Gibco, Rockville, MD, United States) and then seeded into plastic culture dishes without coated tissues at a density of 1 × 10^5^ cells/cm^2^.

Next, the cells were subjected to conventional culture or neuronal differentiation. For conventional culture, NSC cells were seeded in 100 mm Dulbecco’s modified Eagle’s medium (DMEM)/F12 medium (Welgene, Daegu, South Korea) supplemented with N-2, B27 supplement (Gibco-Invitrogen, Carlsbad, CA, United States), 20 ng/ml EGF, and basic fibroblast growth factor (bFGF, BD Biosciences, United States). EGF and bFGF were added every 2 days, and the medium was placed in a 37°C, 5% CO_2_ incubator (Thermo Fisher Scientific, Waltham, MA, United States). Moreover, for the collection of primary neurospheres, the cells were incubated with Accumax cell detachment reagent (Millipore Corp., Bedford, MA, United States). For differentiation, the detached cells were seeded to the plate added with 0.2 mg/ml poly-L-ornithine and 1 μg/ml fibronectin (Sigma-Aldrich) without growth factors or ciliary neurotrophic factor (CNTF, BD Biosciences, San Jose, CA, United States).

For cell transfection, NSCs in the logarithmic growth phase were trypsinized, seeded into six-well plates at a density of 1 × 10^5^ cells/well, and then routinely cultured for 24 h. Afterward, the GV342 vector was enzymatically cleaved by *Age*I, and the gene fragments were ligated to the linearized GV342 vector by ligase. The recombinant positive clones were subjected to DNA sequencing analysis by Invitrogen Corporation (Carlsbad, CA, United States). Upon cell confluence reaching 75%, NSCs were transduced with recombinant lentivirus vectors. The lentiviral vector was purchased from Shanghai GeneChem Co., Ltd. (Shanghai, China). NSCs were transduced with lentiviral vectors carrying plasmids containing short hairpin RNA (shRNA) targeting different sequences of PINK1 (sh-PINK1-1, sh-PINK1-2, and sh-PINK1-3) or negative control shRNA (sh-NC) (Supplementary Table 1). After transduction for 48 h, reverse transcription quantitative polymerase chain reaction (RT-qPCR) was employed to measure the transduction efficiency of the three sh-PINK1 plasmids.

Moreover, in order to validate the effects of miR-421 on self-renewal of human embryonic NSCs, NSCs were respectively transfected with plasmids carrying the miR-421 inhibitor, NC inhibitor, miR-421 mimic, and NC mimic. Besides, in order to validate that miR-421 participates in modulating human embryonic NSCs, NSCs were separately transfected with the plasmids overexpressing miR-421 alone or in combination with PINK1. To investigate whether PINK1 affected FOXO3 by regulating HDAC3, NSCs were separately transfected with the PINK1 overexpression plasmids in combination with RGFP966 (an inhibitor of HDAC3, 3.5 μg/ml, Selleckchem, Houston, TX, United States) or dimethyl sulfoxide (DMSO). To validate that miR-421 boost NSC self-renewal through FOXO3, NSCs were, respectively, transfected with the plasmids expressing miR-421 mimic alone or in combination with SC97 (an inhibitor of FOXO3).

### MiR-421 Mimic/Inhibitor Plasmid Construction and Lentivirus Transduction

The sequence of miR-421 was obtained from the miRWalk database^2^, and the primer sequence for miR-421 mimic/inhibitor was 5′-AUCAACAGACAUUAAUUGGGC GC-3′. Mimic forward sequence: 5′-ATCAACAGACATTAA TTGGGCGC-3′; mimic reverse sequence: 5′-TAGTTGTCTG TAATTAACCCGCG-3′. Inhibitor sequence: 5′-GCGCCCAAU UAAUGUCUGUUGAU-3′. Additionally, the GV342 vector was digested by *Age*I, and then the miR-421 mimic/inhibitor gene fragment was ligated to the linearized GV342 vector by ligase. The DNA sequencing analysis of recombinant positive clones was performed by Invitrogen Corporation (Carlsbad, CA, United States). Furthermore, the recombinant lentivirus was transduced to NSCs, and then the lentivirus was removed after transduction for 24 h. Next, following incubation with a new complete medium for 3 days, the expression of enhanced green fluorescent protein (EGFP) was observed. The transduction efficiency was examined by RT-qPCR. The lentiviral vector was purchased from Shanghai GeneChem Co., Ltd. (Shanghai, China).

### Quantification of Gene Expression

Total RNA was extracted by RNeasy Mini Kit (Qiagen, Valencia, CA, United States). The reverse transcription kit (RR047A, Takara, Tokyo, Japan) was used for the reverse transcription from mRNA to cDNA, and the miRNA First Strand cDNA Synthesis (Tailing Reaction) kit (B532451-0020, Sangon Biotech, Shanghai, China) was adopted to reversely transcribe miRNA to cDNA. Afterward, based on the instructions of SYBR^®^ Premix Ex Taq^TM^ II (Perfect Real Time) kit (DRR081, Takara, Tokyo, Japan), the RT-qPCR reaction was performed by a real-time fluorescent quantitative PCR system (ABI 7500, Applied Biosystems, Foster City, CA, United States). Primers were synthesized by Sangon Biotech Co., Ltd. (Shanghai, China) and are listed in Supplementary Table 2. Glyceraldehyde-3-phosphate dehydrogenase (GAPDH) and U6 were used as internal references for genes and miRNAs. The relative expression of genes was calculated by 2^–ΔΔCt^ method.

### Protein Extraction and Immunoblotting

Cells were lysed with the enhanced radio-immunoprecipitation assay (RIPA) lysis buffer (Wuhan Boster Biological Technology, Ltd., Wuhan, Hubei, China) containing protease inhibitors. After protein concentration quantitation using the bicinchoninic acid (BCA) kit (Boster), the protein was separated by 10% sodium dodecyl sulfate-polyacrylamide gel electrophoresis (SDS-PAGE) and then electro-transferred onto the polyvinylidene fluoride (PVDF) membrane. Next, the membrane was blocked with 5% bovine serum albumin (BSA) for 2 h at room temperature to block non-specific binding. Following this, the blots were probed with a diluted primary antibody (rabbit anti-MSI1, ab52865, 1:1,000; rabbit anti-FOXO3, ab32389, 1:1,000; rabbit anti-HDAC3, ab32369, 1:1,000; rabbit anti-p-HDAC3, ab61056, 1:1,000; rabbit anti-HES1, ab221788, 1:1,000; rabbit anti-PINK1, ab23707, 1:2,000; rabbit anti-BMI1, ab14389, 1:1,000; rabbit anti-MKI-67, ab209897, 1:1,000) at 4°C overnight. After probing with a horseradish peroxidase (HRP)-labeled goat anti-rabbit secondary antibody (ab205719, 1:2,000) at room temperature for 1 h, the blots were visualized with the enhanced chemiluminescence (ECL) working solution (EMD Millipore Corporation, Billerica, MA, United States). The abovementioned antibodies were all purchased from Abcam (Cambridge, United Kingdom). The protein band was quantified by ImageJ software with β-actin as an internal reference.

### Neurosphere Assay

Neurospheres grown in a stem cell-selective medium were dissociated into single cells with a non-enzymatic cell dissociation solution (C5789, Sigma-Aldrich). The Trypan Blue method was adopted to exclude dead cells, and then the live cells were counted. Subsequently, the NSCs were seeded into 96-well plates with the stem cell-selective medium at a density of one to two cells/mm. Following 6–8 days, the newborn neurospheres were observed under the microscope, with quantity and diameter measurement.

### 5-Ethynyl-2′-Deoxyuridine (EdU) Labeling Assay

Cells in the logarithmic growth phase were plated into 96-well plates at a density of 4 × 10^3^–1 × 10^5^ cells/well and cultured to the normal growth stage. Afterward, these cells were incubated with 100 μl 50 μM EdU medium for 2 h, and then after phosphate-buffered saline (PBS) washes, each well was added with a cell fixative (PBS containing 4% paraformaldehyde) and placed at room temperature for 30 min. Moreover, cells were, respectively, treated with glycine, penetrant (PBS containing 0.5% Triton X-100), 1 × Apollo^®^ dying solution, and 1 × Hoechst 33342 reaction solution.

### Neuron Differentiation Assay

For differentiation, dissociated cells were seeded on plates coated with 0.2 mg/ml poly-L-ornithine and 1 μg/ml fibronectin (Sigma-Aldrich). Then, 10 nM miR-421 mimic or mimic-NC was added to incubate cells for 5 days (Choi et al., 2016b). Following this, the expression of marker genes of interest was determined by western blot.

### Dual Luciferase Reporter Gene Assay

The target site wild-type (WT) sequence of the PINK1 3′-untranslated region (3′-UTR) and its site-directed mutation sequence (MUT, sequence: 5′-CCGTCA-3′) were synthesized. The restriction endonuclease was adopted to digest the pGL3-BASIC plasmid (Guangzhou Ribio Biotechnology Co., Ltd., Guangzhou, China), and then the synthesized target gene fragments WT and MUT were inserted into the pmiR-RB-REPORTTM vector (Ribio). Next, the empty plasmid was transfected as the control group, and the correctly sequenced luciferase reporter plasmid was used for subsequent transfection. The vectors were separately transferred with miR-421 mimic to NSCs. After 48 h of transfection, the cells were collected, lysed, and centrifuged for 5 min. Then, the relative light unit (RLU) value of the supernatant was examined by the luciferase detection kit (RG005, Beyotime Biotechnology, Shanghai, China). The degree of activation of the target reporter gene was compared, based on the ratio of the RLU value of β-cal luciferase activity to the RLU value of firefly luciferase activity.

### Co-immunoprecipitation (Co-IP) Assay

Cells were lysed in lysis buffer [50 mM Tris–HCl (pH 7.4), 150 mM NaCl, 10% glycerol, 1 mM EDTA, 0.5% NP-40, and protease inhibitor cocktail], and cell debris is removed by centrifugation. Following this, the supernatant cell lysate was mixed and incubated with anti-Pan Acetylation (66289-1-Ig, 1:100, Proteintech, Wuhan, Hubei, China)/anti-PINK1 (ab216144, 1:200, Abcam, Cambridge, United Kingdom)/anti-HDAC3 (ab219376, 1:200, Abcam, Cambridge, United Kingdom) antibody and protein A/G beads (Santa Cruz Biotechnology, Dallas, TX, United States) for 2 h. Next, after washing with lysis buffer, the precipitate was collected, added with reducing sample buffer, and boiled at 100°C for 5 min. Further, the protein was separated by SDS-PAGE and transferred to PVDF membrane (Millipore, Temecula, CA, United States). Western blot was conducted to assess the degree of FOXO3 acetylation.

### Immunofluorescence Assay

The immunofluorescence assay of NSCs was performed using anti-MKI-67 (ab15580, 1:500, Abcam, Cambridge, United Kingdom), MAP2 (1:500, ab183830), TUJ-1 (1:500, ab14545), GFAP (1:500, ab7260), CNPase (1:500, ab201679), Brdu (1:250, ab6326), and secondary antibody goat anti-rabbit IgG H&L (Cy3^®^, 1:100, ab97075) or goat anti-mouse IgG H&L (Alexa Fluor^®^ 488) (ab150113). The nucleus was stained with DAPI. The images were captured by an upright microscope (DMRB, Leica, Wetzlar, Germany) and processed in Adobe Photoshop CS 5.1. For the quantification of KI-67^+^ (or Bcl-2 or Bax) and BrdU^+^ cells, 36 representative images were obtained and the section was sampled. ImageJ software was used to count the number of positive cells.

### Establishment of IBI Animal Models

A total of 140 7-day-old newborn mice, weighing 4.0 ± 0.5 g, were sham-operated (*n* = 20) or modeled with IBI followed by transduction of lentiviral vectors carrying plasmids expressing miR-421 mimic alone or in combination with SC97 (4 μg/ml, Selleckchem, Houston, TX, United States), overexpression (oe)-PINK, or oe-PINK + oe-FOXO3, *n* = 20. The IBI model was established as previously described (Ten et al., 2003; Kallakuri et al., 2018). Briefly, all mice were housed under controlled conditions of temperature (24°C), with free access to food. All feed and water were sterilized. The mice were briefly anesthetized with isoflurane, with the neck routinely disinfected. Then, the right common carotid artery was ligated after local anesthesia with 2% lidocaine. After recovery for 2 h, the mice were placed at an airtight hypoxia box at 37°C with inhalation of the mixture of 8% oxygen and 92% nitrogen for 30 min. Next, each mouse was subjected to 100 U hyaluronidase through the nose and then intranasally treated with mimic NC, miR-421 mimic, or SC97. At the end of the animal experiment, mice were euthanized by anesthesia and cervical dislocation. In addition, 12 mice of each group were selected for observation of survival conditions at 0, 3, 7, and 9 days after intranasal administration. Besides, eight mice from each group were taken for animal neurobehavioral experiments, and then their brain tissues were excised for further staining and molecular biology experiments.

### Evaluation of Short-Term Mouse Neurobehaviors

The negative geotaxis test was adopted to assess the labyrinthine reflex, which could reflect the strength and co-ordination of mice. Briefly, before the mice were euthanized, sham-operated mice and IBI mice treated with miR-421 mimic alone or in combination with SC97, oe-PINK, or oe-PINK^+^ oe-FOXO3 were subjected to neurobehavioral analysis. Subsequently, the mice were placed in a board with 45° incline, and their heads faced downward. Then, the time of these mice to make a 180° turn and start to climb up to the hill was recorded, and time was limited in 30 s. If the mice failed to complete this task in time, 30 s was the recorded as the result.

### Cerebral Infarction Area in Mice

After euthanizing the mice, its brain tissues were carefully peeled off and placed in the brain trough within 10 min with the cerebellum, olfactory bulb, and lower brain stem removal. Following storage in the −80°C refrigerator for 20 min, 1-mm-thick coronary brain slices were stained with 2% TTC solution (Sigma-Aldrich) at room temperature for 30 min, with gentle shaking every 5 min. Then, the slices were washed with PBS and photographed. Living brain tissues were bright red, while ischemia, infarction, and necrotic tissues were pale. ImageJ software was employed to measure the infarct area and total area of each slice.

### Nissl Staining of Brain Neurons

The frozen sections of brain tissue with a thickness of 10 μm were, respectively, immersed in chloroform, absolute ethanol, 95% ethanol, and 70% ethanol for 1 min each. After washing with distilled water, the sections were incubated in Cresyl violet acetate for 30 min. Then, the sections were differentiated in 95% ethanol, dehydrated, permeabilized, mounted, and observed under a microscope. The Nissl body was purple, and the nucleus and glial cells were orchid.

### TUNEL Assay for Brain Tissues

TUNEL staining was adopted to evaluate cell death. After deparaffinization, hydration, and permeation, the brain tissue sections were added with a TUNEL reaction solution (Roche, Alameda, CA, United States). TUNEL-positive cells were imaged and counted under the microscope (Eclipse Ti-U, Nikon, Tokyo, Japan).

### Statistical Analysis

The data in the present study were processed using SPSS 21.0 software (IBM Corp., Armonk, NY, United States). The measurement data were expressed as mean ± standard deviation. The unpaired *t-*test was employed to analyze unpaired data. The data among multiple groups were compared by one-way analysis of variance (ANOVA) with Tukey’s *post hoc* test. The data at different time points were compared by repeated-measures ANOVA. The survival rate of patients was analyzed by the Kaplan–Meier method. Statistical significance was defined at a *p*-value less than 0.05.

## Results

### MiR-421 Was Downregulated During the Differentiation of Human Embryonic NSCs

**FIGURE 1:**
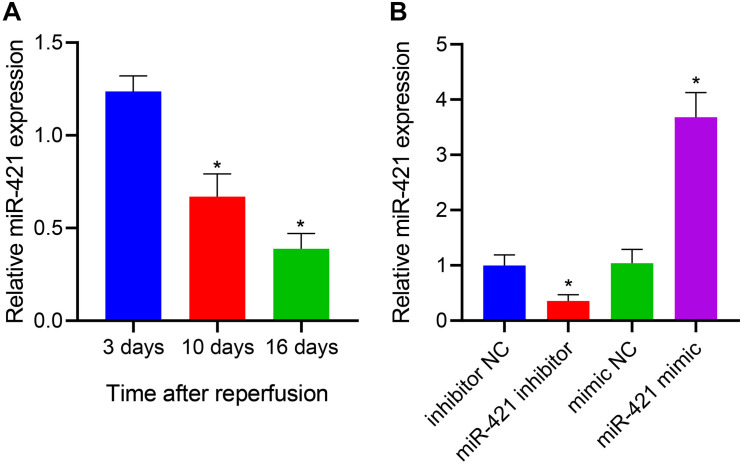
Establishment of lentiviral transduction system. **(A)** The expression of miR-421 before and after stem cell differentiation, as measured by RT-qPCR. **(B)** The level of miR-421 in the presence of miR-421 inhibitor or miR-421 mimic, as measured by RT-qPCR. *p < 0.05 compared with the 3 days group or inhibitor NC group or mimic NC group. Measurement data were summarized as mean ± standard deviation. Data comparison among multiple groups was analyzed by one-way ANOVA with Tukey’s *post hoc* test. Cell experiments were repeated for three times.

miR-421 was highly expressed in the amygdala and played a role in repair after brain injury (Balakathiresan et al., 2014). More importantly, miR-421 overexpression blocked the apoptosis and autophagy of hippocampal neurons in the epilepsy model mice (Wen et al., 2018). Thus, we enhanced and restricted miR-421 expression in human embryonic NSCs by lentiviral transduction and further explored its function. The human embryonic NSCs were subcultured for 7 days, and the neurospheres were digested and seeded in a 48-well plate coated with poly-L-ornithine solution and Laminin at a density of 5 × 10^4^ cells/well. After culturing in a complete medium for 3 days/10 days/16 days, the cells in these three time periods were collected for RT-qPCR detection. As shown in Figure 1A, the results of RT-qPCR demonstrated that relative to human embryonic NSCs, the expression of miR-421 gradually diminished after stem cell differentiation over time. Furthermore, RT-qPCR data revealed that the level of miR-421 was markedly restricted in response to the miR-421 inhibitor, while it was obviously boosted in the presence of the miR-421 mimic (Figure 1B). The above results illustrated that the lentiviral transduction system of human embryonic NSCs was successfully established.

### MiR-421 Overexpression Enhances the Self-Renewal of Embryonic NSCs

**FIGURE 2:**
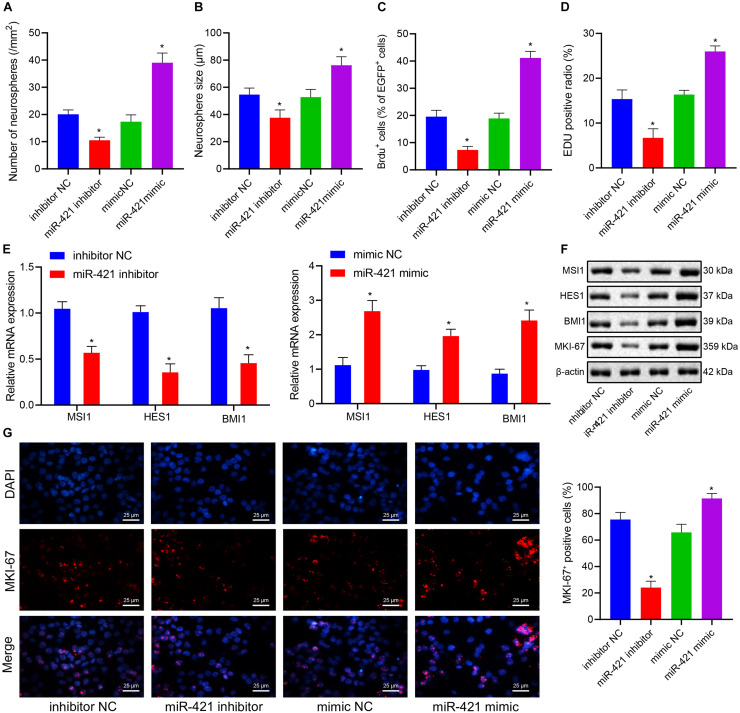
Effects of miR-421 knockdown and overexpression on the self-renewal of human embryonic NSCs. **(A,B)** Number and diameter of neurospheres in the presence of miR-421 inhibitor **(A)** and miR-421 mimic **(B)**. **(C)** The impact of miR-421 inhibitor and miR-421 mimic on cell proliferation by BrdU immunofluorescence staining. **(D)** The proliferation ability of NSCs, as measured by EdU assay. **(E)** The mRNA level of stem genes MSI1, HES1, and BMI1. **(F)** The protein level of stem genes MSI1, HES1, and BMI1, as measured by western blot. **(G)** The expression of MKI-67, as evaluated by immunofluorescence, in response to miR-421 inhibitor or miR-421 mimic. *p < 0.05 compared with mimic NC or inhibitor NC. The experimental data were represented as mean ± standard deviation. The results between two groups were analyzed by the unpaired *t*-test. Data comparison among different groups was analyzed by one-way ANOVA with Tukey’s *post hoc* test. Cellular experiments were repeated for three times.

Self-renewal ability was one of the basic biological characteristics of NSCs, and we moved to investigate whether miR-421 participates in mediating the self-renewal of human embryonic NSCs. Neurosphere assay results indicated that the number and diameter of neurospheres were reduced in response to silencing miR-421 (miR-421 inhibitor), while they were increased upon miR-421 overexpression (miR-421 mimic) (Figures 2A,B). In addition, the results of immunofluorescence staining and EdU assays revealed that miR-421 inhibition restricted NSC proliferation while miR-421 overexpression boosted NSC proliferation (Figures 2C,D). Moreover, RT-qPCR and western blot data revealed that miR-421 overexpression increased the expression of marker genes (MSI1, HES1, and BMI1) of NSCs and proliferation-related gene MKI-67. Conversely, miR-421 knockdown reversed the effects of miR-421 overexpression (Figures 2E,F). Consistently, the immunofluorescence assay also validated the impact of miR-421 on MKI-67 (Figure 2G). Meanwhile, the levels of the markers of neurons (MAP2 and TUJ-1), astrocytes (GFAP), and oligodendrocytes (CNPase) were determined after induction of NSC differentiation. The results showed that on the fifth day of induction, the expression of MAP2, TUJ-1, GFAP, and CNPase was elevated in cells of each group, and the elevation was sharper in the presence of miR-421 overexpression (Supplementary Figure 1A). As shown in Supplementary Figures 1B,C, miR-421 overexpression increased the number and diameter of neurospheres. The results of the BrdU assay presented an enhancement in the number of BrdU^+^ cells following overexpression of miR-421 (Supplementary Figure 1D). The results of the EdU assay displayed an increase of cell proliferation in the presence of overexpression of miR-421 (Supplementary Figure 1E). Taken together, these results suggested that overexpression of miR-421 could promote the self-renewal of NSCs.

### PINK1 Is a Target Gene of MiR-421 in Human Embryonic NSCs

**FIGURE 3:**
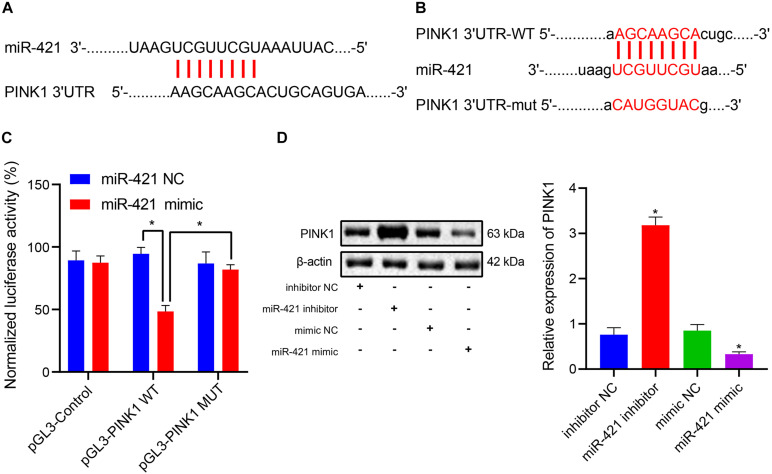
miR-421 targets PINK1 and inhibits its expression in human embryonic NSCs. **(A)** The possible binding site of miR-421 and PINK1 3′-UTR. **(B)** The sequence of PINK1 3′-UTR WT and PINK1 3′-UTR MUT. **(C)** The binding of miR-421 to PINK1 confirmed by dual luciferase reporter assay. **(D)** The expression of PINK1 in the presence of miR-421 inhibitor or miR-421 mimic. *p < 0.05 compared with the inhibitor NC group or mimic NC group. Measurement data were represented as mean ± standard deviation. Data in **(C)** was analyzed by repeated-measures ANOVA. Data of multiple groups in **(D)** were compared by one-way ANOVA with Tukey’s *post hoc* test. Cell experiments were repeated for three times.

During brain development and NSC differentiation, PINK1 deficiency could lead to defects of GFAP-positive astrocytes (Choi et al., 2016a). In the current study, we predicted that miR-421 might bind to 3′UTR of PINK1 (Figures 3A,B). Dual luciferase reporter assay results clarified that the luciferase activity of pGL3-PINK1 WT was suppressed following transfection with miR-421 mimic, which did not affect the luciferase activity of pGL3-PINK1 MUT (Figure 3C), indicating that miR-421 can specifically target PINK1 and regulate its expression. Moreover, the expression of PINK1 was appreciably enhanced after treatment with the miR-421 inhibitor, while PINK1 expression was markedly restricted in response to miR-421 overexpression (Figure 3D). Altogether, miR-421 could target PINK1 and reduce its expression in human embryonic NSC.

### MiR-421 Upregulation Augments the Self-Renewal and Proliferation of Human Embryonic NSCs Through PINK1

**FIGURE 4:**
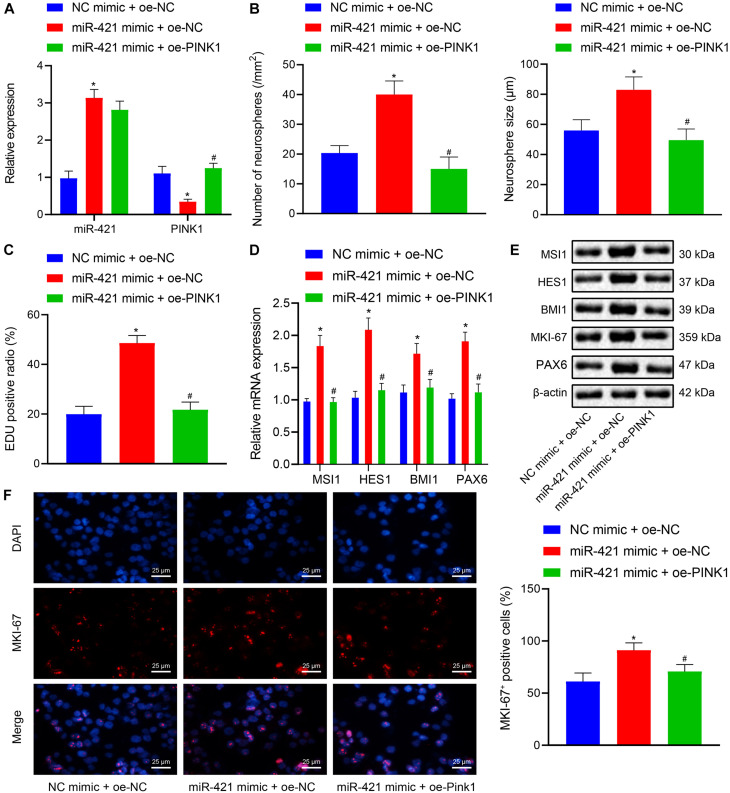
Effects of miR-421 overexpression and PINK1 overexpression on NSC self-renewal and proliferation of human embryonic NSCs. **(A)** The expression of miR-421 and PINK1 in the presence of miR-421 mimic alone or in combination with PINK1 overexpression, as measured by RT-qPCR. **(B)** The statistics of number and diameter of neurospheres. **(C)** NSC proliferation ability in response to miR-421 mimic alone or in combination with PINK1 overexpression, as measured by EdU assay. **(D)** The mRNA level of stem genes (MSI1, HES1, and BMI1) after treatment with miR-421 mimic alone or in combination with oe-PINK1, as measured by RT-qPCR. **(E)** The protein levels of MSI1, HES1, and BMI1, as tested by western blot. **(F)** Effect of miR-421 mimic alone or in combination with PINK1 overexpression on MKI-67 expression, as evaluated by immunofluorescence assay. *p < 0.05 compared with the mimic NC + oe-NC group; ^#^*p* < 0.05 compared with the miR-421 mimic + oe-NC group. Measurement data were summarized as mean ± standard deviation. Data comparison among multiple groups was analyzed by one-way ANOVA with Tukey’s *post hoc* test. Cell experiments were repeated for three times.

The aforementioned experiments had demonstrated that miR-421 modulated the PINK1 expression in human embryonic NSCs, and we moved to validate that miR-421 was involved in regulating NSC self-renewal through PINK1. As shown in Figure 4A, RT-qPCR results suggested that the miR-421 level was enhanced and the PINK1 level was restricted in response to miR-421 mimic alone, while miR-421 expression displayed no change and PINK1 expression was boosted after co-overexpression of miR-421 and PINK1. Besides, miR-421 mimic alone augmented NSC neurosphere-forming ability, NSC proliferation ability, and the expression of stem genes (MSI1, HES1, and BMI1) and proliferation-related gene MKI-67, while the co-overexpression of miR-421 and PINK1 reserved the effects of miR-421 mimic alone (Figures 4B–F). Taken together, these results illuminated that miR-421 stimulated the self-renewal and proliferation of human embryonic NSCs by inhibiting PINK1.

### PINK1 Represses FOXO3 Expression by Accelerating HDAC3 Phosphorylation

**FIGURE 5:**
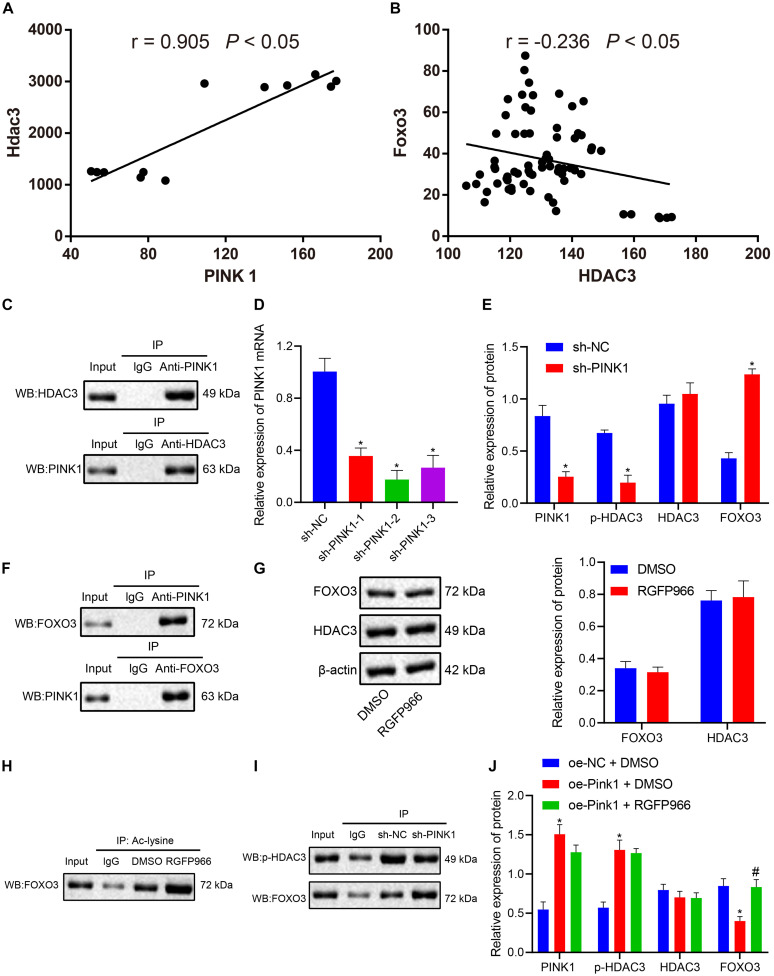
PINK1 accelerates HDAC3 phosphorylation and further restricts FOXO3 expression. **(A)** Correlation of PINK1 expression and HDAC3 expression in NSCs in the GSE143388 dataset. **(B)** Correlation of HDAC3 expression and FOXO3 expression in NSCs in the GSE134688 dataset. **(C)** The interaction between HDAC3 and PINK1 in NSCs, as examined by Co-IP assay and western blot. **(D)** Interference of different sh-PINK1-1, sh-PINK1-2, and sh-PINK1-3 sequences on PINK1 expression in NSCs, as measured by RT-qPCR. **(E)** The expression of PINK1, p-HDAC3, HDAC3, and FOXO3 in response to PINK1 silencing, as measured by western blot. **(F)** The interaction between PINK1 and FOXO3 in NSCs with PINK1 knockdown, as examined by Co-IP assay. **(G)** The protein level of HDAC3 and FOXO3 in NSCs after RGFP966 treatment, as measured by western blot. **(H)** The acetylation level of FOXO3 in the presence of RGFP966 treatment, as detected by Co-IP assay. **(I)** The interaction between p-HDAC3 and FOXO3, as detected by Co-IP assay and western blot. **(J)** The protein levels of PINK1, p-HDAC3, HDAC3, and FOXO3 after treatment with the oe-PINK1 plasmid alone or in combination with RGFP966 treatment, as measured by western blot. *p < 0.05 compared with the sh-NC group or DMSO group or oe-NC + DMSO group. ^#^*p* < 0.05 compared with the oe-PINK1 + DMSO group. The measurement data were summarized by mean ± standard deviation. Results between two groups were compared by the unpaired *t*-test. Data comparison among multiple groups was analyzed by one-way ANOVA with Tukey’s *post hoc* test.

Correlation analysis of the expression data of PINK1 and HDAC3 in the GSE143388 dataset revealed a positive correlation between PINK1 expression and HDAC3 expression in NSCs (Figure 5A). Evidence has shown that NSCs are particularly vulnerable to FOXO3-induced apoptosis (Schmidt-Strassburger et al., 2012). HDAC3 has been reported to form a complex with FOXO3, which reduces the degree of acetylation of FOXO3 and affects its transcriptional activity (Zhang et al., 2017). At the same time, we found that FOXO3 and HDAC3 were negatively correlated with each other in NSCs in the GSE134688 dataset (Figure 5B). We further explored whether the inhibitory effect of PINK1 on FOXO3 transcription was linked to HDAC3 phosphorylation. As shown in Figure 5C, the results of Co-IP and western blot illustrated an interaction between HDAC3 and PINK1 in NSCs. Afterward, RT-qPCR results confirmed that the sh-PINK1-2 sequence had the superior silencing efficiency and was thus selected for subsequent experiments (Figure 5D). After treatment with sh-PINK1, the expression of PINK1 and p-HDAC3 was suppressed, and the HDAC3 level showed no change, while FOXO3 expression was enhanced (Figure 5E). Moreover, Co-IP assay and western blot clarified that PINK1 could interact with FOXO3 in NSCs (Figures 5F,G). RGFP966 was a selective inhibitor of HDAC3 (Wells et al., 2013). RGFP966 treatment did not cause significant change to the total protein level of HDAC3 and FOXO3, as detected by western blot (Figure 5G). Through the enrichment of Ac-lysine, we uncovered that FOXO3 acetylation level markedly boosted following RGFP966 treatment (Figure 5H). Additionally, PINK1 silencing reduced p-HDAC3 expression and augmented FOXO3 expression, suggesting that the interaction between p-HDAC3 and FOXO3 was weakened in NSCs in the context of PINK1 knockdown (Figure 5I). Furthermore, in response to PINK1 overexpression, the expression of PINK1 and p-HDAC3 was enhanced, and FOXO3 and HDAC3 expression showed no obvious change. Moreover, the combination of PINK1 overexpression and RGFP966-induced HDAC3 inhibition did not cause significant difference to FOXO3, PINK1, and HDAC3 levels (Figure 5J). In summary, our data indicated that PINK1 curbed the acetylation and expression of HDAC3 by boosting HDAC3 phosphorylation.

### MiR-421 Facilitates NSC Self-Renewal *in vitro* via the PINK1/HDAC3/FOXO3 Axis

**FIGURE 6:**
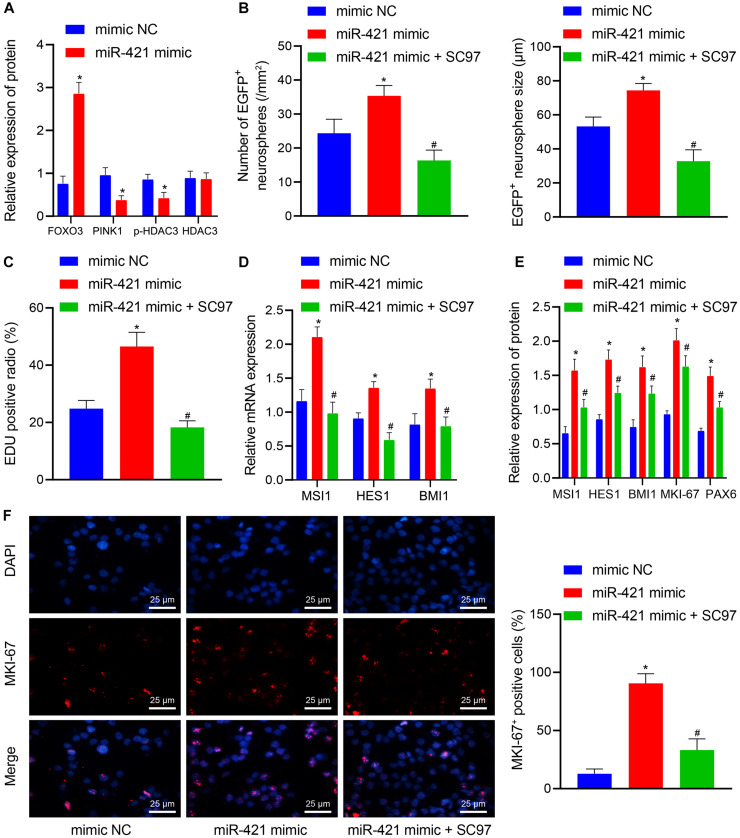
miR-421 enhances NSC self-renewal by mediating the PINK1/HDAC3/FOXO3 axis. **(A)** The protein levels of FOXO3, PINK1, p-HDAC3, and HDAC3 in response to miR-421 mimic, as measured by western blot. **(B)** After treatment with miR-421 mimic or in combination with SC97, the statistics of the number and diameter of neurospheres. **(C)** The proliferation ability of NSCs in different groups, as examined by EdU assay. **(D)** The mRNA level of stem genes MSI1, HES1, and BMI1 in the presence of miR-421 mimic or in combination with SC97, as examined by RT-qPCR. **(E)** The expression of MSI1, HES1, BMI1, and MKI-67 in each group, as measured by western blot. **(F)** Immunofluorescence was adopted to detect the expression of proliferation-related antigen MKI-67. *p < 0.05 compared with the mimic NC group. ^#^*p* < 0.05 compared with the miR-421 mimic group. Measurement data were shown as mean ± standard deviation. The unpaired *t*-test was adopted to analyze the data of two groups. Data comparison among multiple groups was analyzed by one-way ANOVA with Tukey’s *post hoc* test. Cell experiments were repeated in triplicate.

Next, lentiviral miR-421 mimic plasmids were transduced into NSCs to verify the mechanism of the miR-421-mediated PINK1/HDAC3/FOXO3 axis in NSC self-renewal. As shown in Figure 6A, miR-421 mimic suppressed the expression of PINK1 and p-HDAC3 and enhanced FOXO3 expression. SC97 was an expression inhibitor of FOXO3 (Liu et al., 2017). miR-421 mimic was found to augment NSCs’ neurosphere-forming ability, proliferation, stem gene (MSI1, HES1, and BMI1) expression, and proliferation-related antigen MKI-67 expression, which could be abrogated by the combination of miR-421 mimic and SC97-induced FOXO3 inhibition (Figures 6B–F). Collectively, these results illuminated that miR-421 overexpression could augment NSC self-renewal through the PINK1/HDAC3/FOXO3 axis.

### MiR-421 Ameliorates IBI *in vivo* Through the PINK1/HDAC3/FOXO3 Axis

**FIGURE 7:**
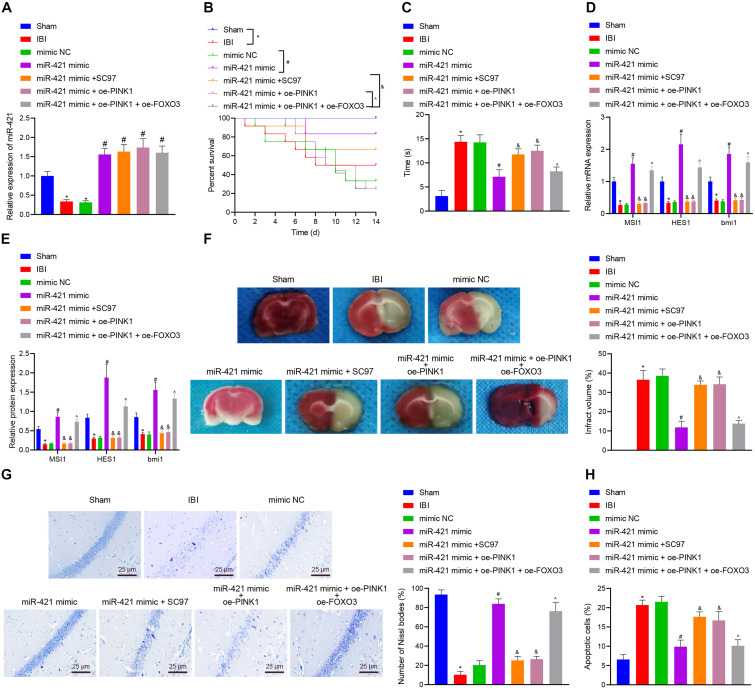
miR-421 relieves IBI *in vivo* by regulating the PINK1/HDAC3/FOXO3 axis. **(A)** The miR-421 expression in the brain tissues of different groups, as measured by RT-qPCR (*n* = 4). **(B)** The survival curves of mice in different treatment groups (*n* = 12). **(C)** The behavioral experiment was adopted to examine the negative geotaxis time of mice (*n* = 8). **(D)** The stem gene expression in mouse brain tissues, as measured by RT-qPCR (*n* = 4). **(E)** The protein level of stem genes in mouse brain tissues of different groups, as detected by western blot (*n* = 4). **(F)** Quantitative TTC staining evaluating the infarct area of mice in each group (*n* = 4). **(G)** Structural changes of Nissl bodies in mouse hippocampus (*n* = 4). **(H)** TUNEL staining was adopted to assess apoptosis in mouse hippocampus (*n* = 4). *p < 0.05 compared with the sham group. ^#^*p* < 0.05 compared with the IBI group. ^&^*p* < 0.05 compared with the miR-421 mimic group. ^*p* < 0.05 compared with the miR-421 mimic + oe-PINK1 group. *n* = 5. Measurement data were summarized as mean ± standard deviation. The data comparison between multiple groups was performed by one-way ANOVA with Tukey’s *post hoc* test. The Kaplan–Meier method was used to calculate the survival rate of mice in **(B)**. Cell experiments were repeated for three times.

In order to investigate whether the miR-421-regulated PINK1/HDAC3/FOXO3 axis participated in self-renewal of nerve cells with IBI, we established a mouse model with IBI to validate the hypothesis. As shown in Figure 7A, relative to sham-operated mice, the expression of miR-421 was reduced in the brain tissues of untreated IBI mice while it was upregulated in brain tissues of IBI mice treated with miR-421 mimic. Moreover, the survival rate of mice within 14 days revealed that in comparison with sham-operated mice, the survival rate was reduced in untreated IBI mice. Treatment with miR-421 mimic reduced the survival rate while an opposite result was noted in the presence of further SC97 or oe-PINK1 treatment. Treatment with miR-421 mimic + oe-PINK1 + oe-FOXO3 resulted in a lower survival rate than treatment with miR-421 mimic + oe-PINK1 (Figure 7B). In addition, compared to sham-operated mice, the negative geotaxis time of untreated IBI mice was prolonged, while it was decreased in miR-421 mimic-treated mice. Additionally, the negative geotaxis time was longer in response to the co-overexpression of miR-421 and PINK1 or miR-421 and SC97 as compared with miR-421 overexpression alone. A reduction was observed in negative geotaxis time of mice treated with miR-421 mimic + oe-PINK1 + oe-FOXO3 (Figure 7C). Moreover, the results of RT-qPCR and western blot revealed that compared with sham-operated mice, the expression of MSI1, HES1, and BMI1 was restricted in the brain tissues of IBI mice. miR-421 mimic increased the expression of MSI1, HES1, and BMI1, which was negated by further oe-PINK1 or SC97 treatment. miR-421 mimic + oe-PINK1 + oe-FOXO3 treatment led to enhanced expression of MSI1, HES1, and BMI1 (Figures 7D,E). Subsequently, TTC staining and HE staining indicated that the cerebral infarcted area of IBI mice was markedly larger than that in sham-operated mice. The cerebral infarcted area was decreased in the miR-421 mimic-treated mice while it was increased in mice treated with miR-421 mimic + oe-PINK1 or miR-421 mimic + SC97. Moreover, the area was reduced upon treatment with miR-421 mimic + oe-PINK1 + oe-FOXO3 (Figure 7F). It had been documented that the hippocampus was the most vulnerable area after IBI (Bartley et al., 2005; Sinha et al., 2018), and thus we conducted Nissl staining on the hippocampus (Figure 7G). The edge of mouse brain tissues of sham-operated mice was clear, with the nucleus in the middle and obvious nucleoli in nerve cells. Besides, Nissl bodies were evenly distributed around the core, and the size and number of Nissl bodies were large. However, in IBI mice, the hippocampus on the ischemic side of the mouse brain was necrotic; the nucleus is obviously atrophic; nuclear fragmentation and cell lysis occurred; and the number of Nissl bodies was significantly reduced or even disappeared. Meanwhile, in the miR-421 mimic-treated mice, there was a small amount of cell necrosis around the hippocampus of mice, and the number of Nissl bodies was partially reduced; compared to miR-421 mimic-treated IBI mice, miR-421 mimic + oe-PINK1- or miR-421 mimic + SC97-treated mice exhibited a larger amount of nuclear necrosis around the hippocampus, nuclear pyknosis, nuclear fragmentation, cell lysis and disappearance, and decreased number of Nissl bodies. Additionally, treatment with miR-421 mimic + oe-PINK1 + oe-FOXO3 reduced the amount of nuclear necrosis around the hippocampus while increasing the number of Nissl bodies. Furthermore, the TUNEL staining illustrated that in comparison with sham-operated mice, the number of apoptotic cells appreciably increased in the IBI mice, while miR-421 mimic markedly suppressed cell apoptosis induced by IBI. Besides, the number of apoptotic cells was increased in the presence of the dual treatment with miR-421 mimic and oe-PINK1 or miR-421 mimic and oe-SC97 versus miR-421 overexpression alone, whereas a contrary result was observed in response to treatment with miR-421 mimic + oe-PINK1 + oe-FOXO3 (Figure 7H). Taken together, these lines of evidence indicate that miR-421 can arrest IBI *in vivo* by regulating the PINK1/HDAC3/FOXO3 axis.

## Discussion

**FIGURE 8:**
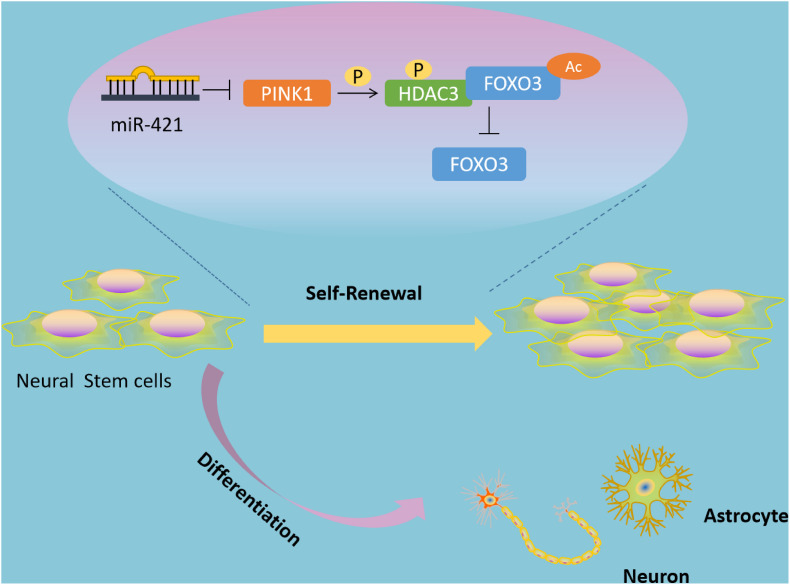
Schematic diagram of the mechanism by which miR-421 modulates the self-renewal of NSCs through the PINK1/HDAC3/FOXO3 axis. miR-421 binds to PINK1 3′-UTR to restrict its transcription expression, further downregulating HDAC3 phosphorylation, and affecting the binding between HDAC3 and FOXO3. This results in upregulation of FOXO3 acetylation and subsequent increase in the expression of FOXO3, thereby enhancing the self-renewal of NSC. Therefore, miR-421 enhances the NSC self-renewal *via* the PINK1/HDAC3/FOXO3 axis.

Self-renewal and multipotent differentiation are the distinctive characteristics of stem cells (Fawal et al., 2018). In the developing brain, the balance of NSC self-renewal and differentiation is critical to ensuring the correct numbers and types of neural cells (Gotz et al., 2016). In addition, emerging lines of evidence have shown that NSCs confer a role in multiple neurological diseases, such as Alzheimer’s disease and IBI (Li et al., 2015; Bernstock et al., 2017). Of note, transplanted NSCs can restore neurological function and recover the secretion of neurotrophic factors, but the main problem that needs to be solved is the limited proliferative and differentiative ability of transplanted NSCs (Zhou et al., 2009; Yang et al., 2014). Researchers have discovered some biomolecules that can facilitate NSC self-renewal *in vitro*, such as tenuigenin and prostaglandin E2 (Liang et al., 2011; Chen et al., 2012; Wong et al., 2014), but these molecules have poor performance in biocompatibility or bioavailability (Ma et al., 2018). Thus, in the current study, we explored the molecular mechanisms underlying the regulatory role of miR-421 in NSC self-renewal (Figure 8), which may provide new therapeutic targets for NSC-related neurological diseases.

It should be noted that miRs are enriched in the nervous system and closely associated with neurogenesis and brain development (Ji et al., 2013; Shu et al., 2013). Emerging evidence suggests that miRs participate in cell differentiation, cell migration, and cell fate determination (Rajman and Schratt, 2017). For example, miR-9 mediates NSC proliferation and differentiation by regulating nuclear receptor TLX and other transcription factors (Zhao et al., 2009; Uchida, 2010); miR-29a plays a role in brain development and damage repair by mediating NSC development and neurite outgrowth (Ma et al., 2020). The interaction between miRs and epigenetic machinery is also linked to multiple NSC-related cellular mechanisms (Lopez-Ramirez and Nicoli, 2014). miR-421, located at X chromosome 13.2, is overexpressed in various diseases, including neuroblastoma, prostate cancer, and pancreatic cancer (Hu et al., 2010; Hao et al., 2011; Ostling et al., 2011). The study of Wen et al. (2018) has highlighted that overexpressed miR-421 could constrain the autophagy and apoptosis of hippocampal neurons. Moreover, Balakathiresan et al. (2014) provided evidence demonstrating that miR-421 was highly expressed in the amygdala and held the potential of repair after brain injury. Further, our experiments confirmed that miR-421 overexpression could augment the self-renewal of NSC, which may participate in the development of a variety of neurological diseases.

Based on gain- and loss-of-function assays, we revealed that miR-421 affected the self-renewal and proliferation of NSC by mediating PINK1. Evidence provided by Wang et al. (2015) has also demonstrated that miR-421 boosted cell apoptosis and mitochondrial fragmentation by inhibiting PINK1 translation. PINK1, a mitochondrial kinase, plays a role in mitochondrial quality control and is related to recessive familial Parkinson’s disease (Valente et al., 2004; Ryan et al., 2015). It has been reported that loss of PINK1 curbed differentiation of newborn neurons in the hippocampus and caused metabolic deficits in adult NSCs (Agnihotri et al., 2017). Choi et al. (2016a) also argued that PINK1 deficiency affected brain development and NSC differentiation by impeding GFAP-positive astrogliogenesis.

Further mechanistic investigation identified that the NSC self-renewal role of PINK1 was also achieved through the HDAC3-FOXO3 axis. HDAC3 is a multimolecular complex containing NCoR and SMRT subunits, which are involved in the physiological effect of multiple nuclear hormone receptors (Zhao et al., 2008; Bonaguidi et al., 2012). Besides, HDAC3 is also recognized as a corepressor for several transcription factors with sequence specificity (Malumbres and Barbacid, 2009; Julian et al., 2013; Pauklin and Vallier, 2013), and HDAC3 inhibitors have been documented to restrict NSC proliferation and facilitate neuronal differentiation (Hsieh et al., 2004). Moreover, HDAC3 is known for its role as an epigenetic mediator of downstream genes through histone deacetylation (Haberland et al., 2009). Notably, Zhang et al. (2017) provided the evidence that the formation of HDAC3-FOXO3 complex blocked the degree of FOXO3 acetylation. FOXO3 has been suggested to regulate the autophagy process in NSCs *via* a network of autophagy genes (Audesse et al., 2019). Furthermore, FOXO3 protects dopaminergic neurons against the accumulation of α-synuclein oligomer (Pino et al., 2014; Angelova et al., 2018), and it also shows the potential to protect neurons in the aging brain (Hwang et al., 2018). Herein, it would be important to further investigate how FOXO3 regulates autophagy in different cell types of brain to offer various targets for different nervous system diseases.

## Conclusion

Based on the results of the current study, miR-421 was poorly expressed in the process of human embryonic NSCs. Moreover, miR-421 could bind to PINK1 3′-UTR and further activate the HDAC3-FOXO3 axis, thus contributing to the self-renewal and proliferation of NSCs. We hope that our findings will pave a way for future therapies against IBI and NSC-related nervous system disorders. However, due to the fact that our experiments were only performed in NSCs and mice, clinical trials with human subjects should be carried out for further studies.

## Data Availability Statement

The original contributions presented in the study are included in the article/Supplementary Material, further inquiries can be directed to the corresponding author/s.

## Ethics Statement

The animal study was reviewed and approved by The Second Xiangya Hospital of Central South University.

## Author Contributions

JJ participated in the conception and design of the study. ML and ZT performed the analysis and interpretation of data. YC contributed to drafting the article. MW and MY revised it critically for important intellectual content. All authors have read and approved the final version of the manuscript.

## Conflict of Interest

The authors declare that the research was conducted in the absence of any commercial or financial relationships that could be construed as a potential conflict of interest.

## Footnotes

1 https://www.ncbi.nlm.nih.gov/gds

2 http://mirwalk.umm.uni-heidelberg.de/human/mirna

## Supplementary Material

Supplementary Figure 1 miR-421 overexpression facilitates the self-renewal of human embryonic NSCs. **(A)** Levels of the markers of neurons (MAP2 and TUJ-1), astrocytes (GFAP) and oligodendrocytes (CNPase) determined by immunofluorescence staining. **(B)** The number of neurospheres in the presence of miR-421 overexpression. **(C)** The diameter of neurospheres in the presence of miR-421 overexpression. **(D)** The number of BrdU^+^ cells following overexpression of miR-421. **(E)** EdU-positive cells in the presence of overexpression of miR-421. ^∗^*p* < 0.05 compared with D0 group.^ #^*p* < 0.05 compared with mimic-NC group. Measurement data were shown as mean ± standard deviation. Unpaired *t*-test was adopted to analyze the data of two groups. Cell experiments were repeated in triplicate.

## Abbreviations

PINK1: PTEN-induced putative kinase 1
FoxO3: Forkhead box O3
NSCs: neural stem cells
IBI: ischemic brain injury
HDAC3: histone deacetylase3
EGF: epidermal growth factor
WT: wide type
MUT: mutant
GEO: Gene Expression Omnibus
Co-IP: Co-immunoprecipitation. 3′-UTR, 3′-untranslated region
BSA: bovine serum albumin
TUNEL: terminal deoxynucleotidyl transferase-mediated dUTP-biotin nick end labeling
PBS: phosphate-buffered saline
RT-qPCR: reverse transcription quantitative polymerase chain reaction
GAPDH: glyceraldehyde-3-phosphate dehydrogenase
RIPA: radio-immunoprecipitation assay
BCA: bicinchoninic acid
SDS-PAGE: sodium dodecyl sulfate polyacrylamide gel electrophoresis
PVDF: polyvinylidene fluoride
HRP: horseradish peroxidase
ECL: enhanced chemiluminescence
BGF: basic fibroblast growth factor
bFGF: basic fibroblast growth factor
CNTF: ciliary neurotrophic factor
shRNA: short hairpin RNA
NC: negative control
EGFP: enhanced green fluorescent protein
EdU: 5-ethynyl-2′-deoxyuridine
oe: overexpression
ANOVA: analysis of variance.
