# Supplementary figures and images for: MiR-421 Binds to PINK1 and Enhances Neural Stem Cell Self-Renewal via HDAC3-Dependent FOXO3 Activation
Source: Front Cell Dev Biol. 2021 Jul 20;9:621187. doi: 10.3389/fcell.2021.621187 (PMC8329493; doi:10.3389/fcell.2021.621187)

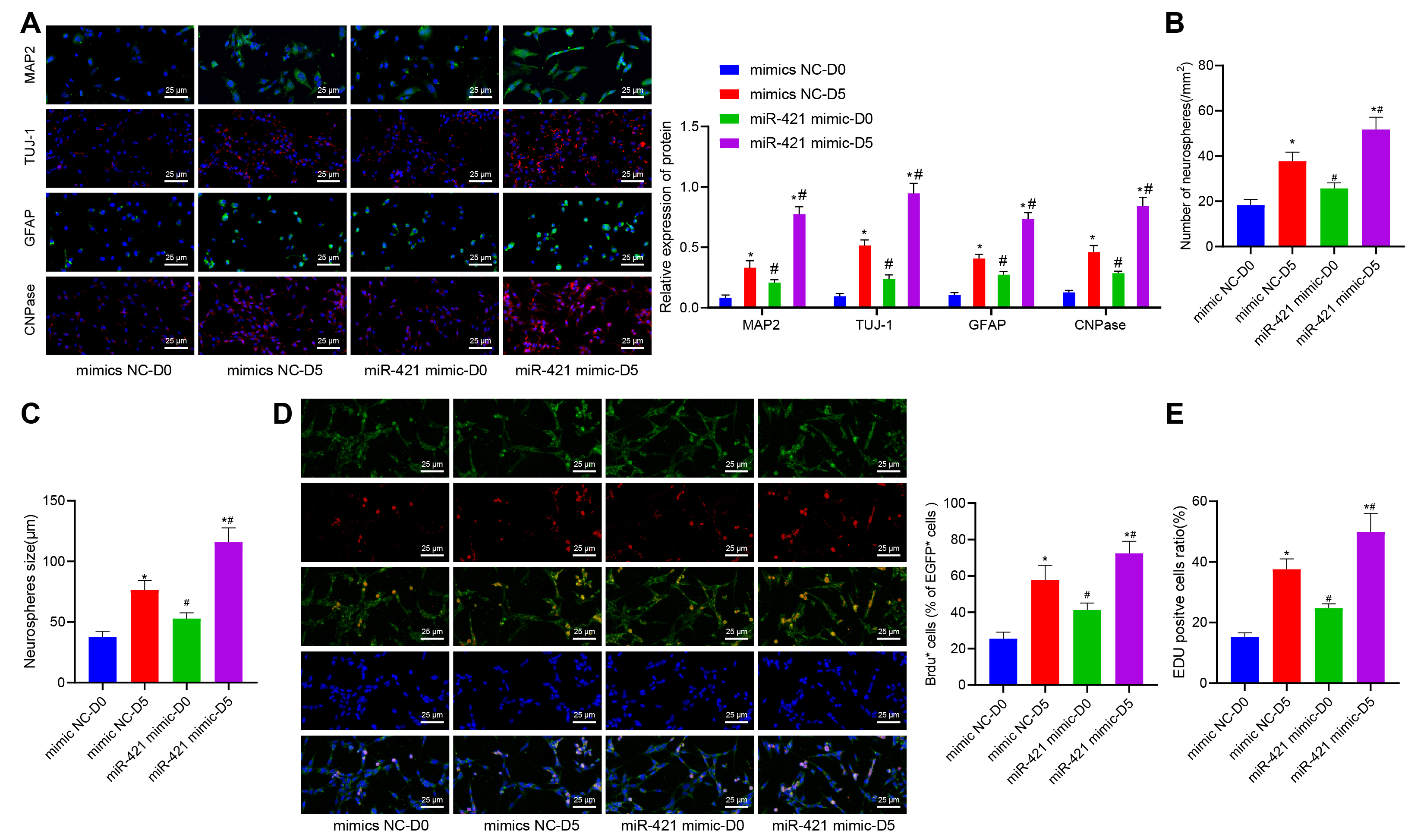

Supplement: Supplementary Figure 1 — miR-421 overexpression facilitates the self-renewal of human embryonic NSCs. (A) Levels of the markers of neurons (MAP2 and TUJ-1), astrocytes (GFAP) and oligodendrocytes (CNPase) determined by immunofluorescence staining. (B) The number of neurospheres in the presence of miR-421 overexpression. (C) The diameter of neurospheres in the presence of miR-421 overexpression. (D) The number of BrdU+ cells following overexpression of miR-421. (E) EdU-positive cells in the presence of overexpression of miR-421. ∗p < 0.05 compared with D0 group. #p < 0.05 compared with mimic-NC group. Measurement data were shown as mean ± standard deviation. Unpaired t-test was adopted to analyze the data of two groups. Cell experiments were repeated in triplicate. [file Image_1.JPEG]
