# Supplementary material for: MiR-421 Binds to PINK1 and Enhances Neural Stem Cell Self-Renewal via HDAC3-Dependent FOXO3 Activation
Source: Front Cell Dev Biol. 2021 Jul 20;9:621187. doi: 10.3389/fcell.2021.621187 (PMC8329493; doi:10.3389/fcell.2021.621187)
Supplement: Supplementary file 2 [file Table_1.DOCX]

**Table S1** Sequences of shRNA (sh).

| shRNAs | Sequence (5′-3′) |
| --- | --- |
| sh-PINK1-1 | GCTAGAGCTTGGTTCAAATGA |
| sh-PINK1-2 | GGTGCATTAAGAATCAGTTAT |
| sh-PINK1-3 | GCATTAAGAATCAGTTATTGC |
| sh-NC | GGGAACUCACGUCAGAA |

Note: PINK1, PTEN induced kinase 1.
