# Supplementary material for: MiR-421 Binds to PINK1 and Enhances Neural Stem Cell Self-Renewal via HDAC3-Dependent FOXO3 Activation
Source: Front Cell Dev Biol. 2021 Jul 20;9:621187. doi: 10.3389/fcell.2021.621187 (PMC8329493; doi:10.3389/fcell.2021.621187)
Supplement: Supplementary file 3 [file Table_2.docx]

**Table S2** Primer sequences for RT-qPCR

| Gene | Sequence |
| --- | --- |
| PINK1 | F: TGTGTCGTGATGGTCTGTGA |
|  | R: CCAGCTTGGCCATTCACTTT |
| GAPDH | F: ATCAAGAAGGTGGTGAAGCA |
|  | R: AAGGTGGAAGAATGGGAGTTG |
| FOXO3 | F: AGTGGATGGTGCGCTGTGT |
|  | R: CTGTGCAGGGACAGGTTGT |
| MSI1 | F: ATGTGGCTCAGCAGGTTGAG |
|  | R: TGCTGCTGTTCACCTTGATGC |
| HES1 | F: TGTCTTGGCCTATATCTGTTC |
|  | R: ACTCTTTCCTCTGGGCTTTGC |
| BMI1 | F: ACTACACCGACACTAATTCCCA |
|  | R: TCCAAAATGGCTCGGAGTCC |
| miR-421 | F: AAACTAATCCGTAAGGGCAA |
|  | R: ACACAGGCATAAATACAGTA |
| U6 | F: GCTTCGGCAGCACATATACT |
|  | R: AACGCTTCACGAATTTGCGT |

Note: F, forward; R, reverse; PINK1, PTEN induced kinase 1; GAPDH, glyceraldehyde-3-phosphate dehydrogenase; FOXO3, forkhead box O3; MSI1, musashi RNA binding protein 1; HES1, hes family bHLH transcription factor 1; BMI1, BMI1 proto-oncogene, polycomb ring finger.
